# Neonatal Hyperglycemia due to Transient Neonatal Diabetes Mellitus in Puerto Rico

**DOI:** 10.1155/2015/984214

**Published:** 2015-10-20

**Authors:** N. Fargas-Berríos, L. García-Fragoso, I. García-García, M. Valcárcel

**Affiliations:** Neonatology Section, Department of Pediatrics, University Pediatrics Hospital, School of Medicine, Medical Sciences Campus, University of Puerto Rico, San Juan, PR 00936-5067, USA

## Abstract

Neonatal hyperglycemia is a metabolic disorder found in the neonatal intensive care units. Neonatal diabetes mellitus (NDM) is a very uncommon cause of hyperglycemia in the newborn, occurring in 1 in every 400,000 births. There are two subtypes of neonatal diabetes mellitus: permanent neonatal diabetes mellitus (PNDM) and transient neonatal diabetes mellitus (TNDM). We describe a term, small for gestational age, female neonate with transient neonatal diabetes mellitus who presented with poor feeding tolerance and vomiting associated with hyperglycemia (385 mg/dL), glycosuria, and metabolic acidosis within the first 12 hours of life. The neonate was treated with intravenous insulin, obtaining a slight control of hyperglycemia. An adequate glycemia was achieved at 5 weeks of life. The molecular studies showed complete loss of maternal methylation at the TND differentially methylated region on chromosome 6q24. The etiology of this neonate's hyperglycemia was a hypomethylation of the maternal TND locus. A rare cause of neonatal diabetes mellitus must be considered if a neonate presents refractory hyperglycemia. To our knowledge, this is the first case reported in Puerto Rico of transient neonatal mellitus due to the uncommon mechanism of maternal hypomethylation of the TND locus. Its prevalence in Puerto Rico is unknown.

## 1. Introduction

Neonatal hyperglycemia is a metabolic disorder found in the neonatal intensive care units [1]. There are several and different etiologies for neonatal hyperglycemia with different clinical pictures and treatments [2].

Neonatal diabetes mellitus (NDM) is a very uncommon cause of hyperglycemia in the newborn, occurring in 1 in every 300,000 to 400,000 live births [1, 3, 4]. NDM presents as insulin requiring persistent hyperglycemia occurring in the first 6 postnatal months, associated with insufficient production of endogenous insulin [1, 3]. NDM is not an autoimmune disorder as insulin-dependent diabetes mellitus in childhood [1, 3]. The insulinopenia of NDM results from abnormal pancreatic islet development, decreased B-cell mass, or B-cell dysfunction [3].

The neonates with NDM are small for gestational age or intrauterine growth retarded and can present with signs of dehydration, weight loss, and glucosuria with or without ketoacidosis or ketonuria. Additionally, they may present with failure to thrive, macroglossia, umbilical hernia, malformations of the brain, heart, or kidneys, hypotonia, deafness, and neurodevelopmental delay [1, 3, 4].

The prevalence of neonatal diabetes mellitus in Puerto Rico is unknown and few cases have been reported [5]. We describe a term, small for gestational age, female neonate with transient neonatal diabetes mellitus in Puerto Rico who presented with poor feeding tolerance and vomiting associated with hyperglycemia, glycosuria, and metabolic acidosis within the first 12 hours of life.

## 2. Case Presentation

A term, small for gestational age, female neonate was born via spontaneous vaginal delivery at 39 weeks of gestational age to a 23-year-old G2P1A0 woman. The mother received adequate prenatal care and prenatal test results indicated that the mother was hepatitis B surface antigen, VDRL, and HIV negative. The mother had no past medical history for systemic illness and she had several urinary tract infections during the pregnancy. The maternal family history was unremarkable for any systemic illness. The paternal family history was significant for hypertension and type 2 diabetes mellitus. There was no history of consanguinity.

The neonate, who weighed 2,041 g (less than 3rd percentile for gestational age), was vigorous and had a spontaneous cry during birth. The initial blood glucose level was 65 mg/dL. The neonate presented with poor feeding tolerance associated with vomiting and was diagnosed with clinical sepsis. Intravenous antibiotics were administered. The blood glucose levels increased to 206–385 mg/dL with the ingestion of milk formula. The neonate was started on continuous intravenous regular insulin infusion at 13 hours of life. Arterial blood gases analysis revealed pH of 7.266, pCO_2_ of 25.6 mm Hg, HCO_3_ 11.8 mmol/L, and base excess −12.7 mmol/L. The insulin infusion was discontinued after adequate glycemic control (104 mg/dL). The neonate was transferred to the Neonatal Intensive Care Unit (NICU) of the University Pediatrics Hospital for evaluation.

**Figure 1:**
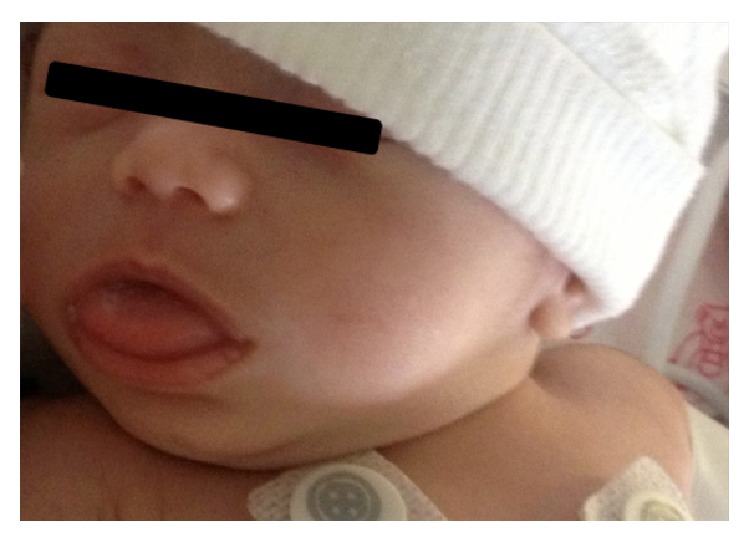
TSGA neonate with macroglossia.

**Figure 2:**
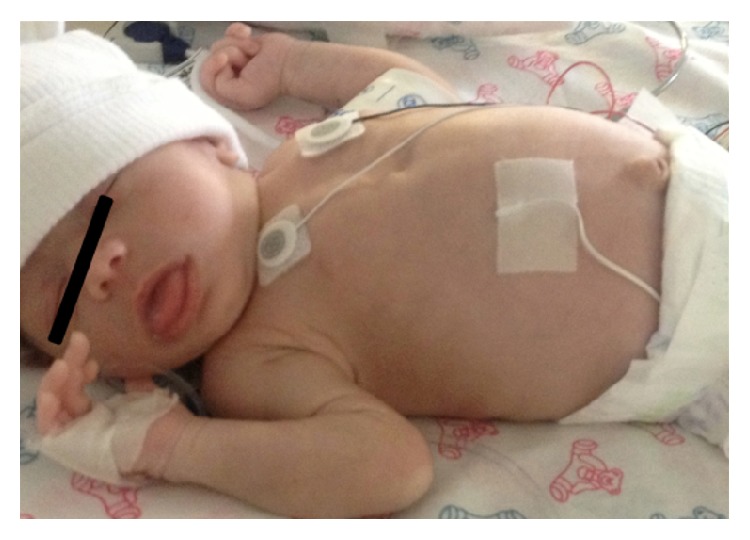
TSGA neonate shows an umbilical hernia.

On admission to the NICU of the University Pediatrics Hospital, physical examination of the infant revealed normal vital signs (temperature of 36.5°C, heart rate of 145 beats/min, respiratory rate of 33 breaths/min, and mean blood pressure of 53 mm Hg), comfortable breathing at room air, macroglossia, and umbilical hernia (Figures 1 and 2). The neonate presented with blood glucose level of 63 mg/dL on admission. The infant was NPO with TPN CHO 5% and Intralipids 20% to maintain adequate hydration and nutritional requirements. Arterial blood gases analysis was normal with pH of 7.412, pCO_2_ of 35.6 mm Hg, HCO_3_ 22.3 mmol/L, and base excess −1.8 mmol/L. The blood glucose levels increased to 320–415 mg/dL and continuous intravenous regular insulin infusion (0.1 units/kg/hr) was started. The carbohydrate infusion was decreased to 3.5 mg/kg/min. Arterial blood gases analysis was performed during the hyperglycemia revealing pH of 7.393, pCO_2_ of 23.9 mm Hg, HCO_3_ 14.2 mmol/L, and base excess −8.5 mmol/L.

The laboratory workup revealed glucosuria (more than 1,000 mg/dL) and ketonuria (trace), normal C-reactive protein, ammonia, lactate, insulin level (1 *μ*U/mL), and thyroid stimulating hormone level. The blood glucose levels ranged from 44 to 314 mg/dL. The insulin infusion was discontinued due to hypoglycemia and the carbohydrate infusion was increased. The endocrinologist recommended insulin lispro injection of 0.5 units if the blood glucose was greater than 200 mg/dL. The blood glucose levels ranged from 46 to 286 mg/dL during the next 3 days.

The abdominal ultrasound was normal, which ruled out any structural disease of the pancreas. The molecular analysis revealed complete loss of maternal methylation at the TND differentially methylated region on chromosome 6q24.

The neonate obtained an adequate glycemia (87 to 118 mg/dL) at 5 weeks of life. The neonate was discharged on enteral feedings with high protein content and without any insulin or hypoglycemic treatment.

## 3. Discussion

The cause of neonatal hyperglycemia must be thoroughly investigated due to its diverse etiologies, clinical pictures, and treatments. The symptomatology is nonspecific and involves diverse neonatal diagnoses, as sepsis. An uncommon etiology as neonatal diabetes mellitus must be considered if a neonate presents refractory hyperglycemia within the first 6 postnatal months and low birth weight [3].

There are two subtypes of neonatal diabetes mellitus: permanent neonatal diabetes mellitus (PNDM) and transient neonatal diabetes mellitus (TNDM) [1, 3]. Transient neonatal diabetes mellitus (TNDM) accounts for 50–60% of NDM cases and is associated with mutations of sulfonylurea receptors at chromosome 6q24 [1, 3, 6]. This subtype presents soon after birth with a spontaneous remission during infancy. The hyperglycemia may begin in the first 6 postnatal weeks in a term infant and usually improves by 24 months [1]. A number of patients may have a relapse to a permanent form of diabetes mellitus in childhood or adolescence [1, 3, 4]. Permanent neonatal diabetes mellitus (PNDM) is less common and is also characterized by early hyperglycemia. This subtype has no period of remission and must be treated lifelong [3].

Three mechanisms are known to cause TNDM in 90% of cases. All mechanisms involve the altered expression of genes in chromosome 6 due to inappropriate overexpression of the chromosome region 6q24. The three mechanisms are (1) paternal uniparental disomy of chromosome 6 (UPD6pat), (2) unbalanced duplication of 6q24 on the paternal allele, and (3) 6q24 maternal hypomethylation defect [1, 3, 4].

TNDM is effectively treated with insulin and oral sulfonylurea medications and has a spontaneous remission within 1 year, but a small number of patients have relapses in adolescence and adulthood [1, 3, 4]. 50% of patients with 6q24-related TNDM develop permanent diabetes mellitus later in life [1]. PNDM is less common than TNDM but requires lifelong treatment [3]. The prompt diagnosis and treatment of neonatal diabetes mellitus are very important because the adequate control of hyperglycemia promotes a satisfactory weight gain and growth.

Blood samples from the neonate and her parents were sent to the Wessex Regional Genetics Laboratory of the University of Southampton in the United Kingdom. The blood samples were tested for methylation-specific polymerase chain reaction (PCR) of deoxyribonucleic acid (DNA). The DNA-PCR test showed complete loss of maternal methylation at the TND differentially methylated region on chromosome 6q24. Further molecular analyses of the polymorphic loci were performed to determine whether the loss of maternal methylation was caused by paternal uniparental disomy of chromosome 6. The inheritance pattern was not consistent with paternal uniparental disomy. The etiology of this neonate's hyperglycemia was a hypomethylation of the maternal TND locus. The neonate was diagnosed with transient neonatal diabetes mellitus (TNDM) due to 6q24 methylation defect.

The prevalence of neonatal diabetes mellitus in Puerto Rico is unknown and few cases have been reported. Two Puerto Rican infants were identified with NDM due to a KCNJ11 activating mutation [5]. To our knowledge this is the first case reported in Puerto Rico with transient neonatal mellitus due to the uncommon mechanism of maternal hypomethylation of the TDN locus.
